# Erratum to: Targeted genotyping‐by‐sequencing of potato and data analysis with R/polyBreedR

**DOI:** 10.1002/tpg2.70011

**Published:** 2025-03-18

**Authors:** Jeffrey B. Endelman, Moctar Kante, Hannele Lindqvist‐Kreuze, Andrzej Kilian, Laura M. Shannon, Maria V. Caraza‐Harter, Brieanne Vaillancourt, Kathrine Mailloux, John P. Hamilton, C. Robin Buell

This erratum corrects the following:

The Greek letters mu and sigma were accidently swapped on page 5, column 1, lines 1 and 5.

The original text reads “between these quantities in a Poisson distribution is 𝜇 = 𝜎^0.5^, which is a straight line with slope 0.5 on a log–log plot (dashed line in Figure 2B). The observed data were overdispersed (i.e., more variable) compared to the Poisson, with a slope 0.79 (SE0.00), meaning that 𝜇 ≈ 𝜎^0.8^.”

It has been corrected to read “between these quantities in a Poisson distribution is 𝜎 = 𝜇^0.5^, which is a straight line with slope 0.5 on a log–log plot (dashed line in Figure 2B). The observed data were overdispersed (i.e., more variable) compared to the Poisson, with a slope 0.79 (SE0.00), meaning that 𝜎 ≈ 𝜇^0.8^.”
